# Single Circular Chromosome Identified from the Genome Sequence of the Vibrio cholerae O1 bv. El Tor Ogawa Strain V060002

**DOI:** 10.1128/genomeA.00564-18

**Published:** 2018-06-21

**Authors:** Shouji Yamamoto, Ken-ichi Lee, Masatomo Morita, Eiji Arakawa, Hidemasa Izumiya, Makoto Ohnishi

**Affiliations:** a Department of Bacteriology I, National Institute of Infectious Diseases, Tokyo, Japan

## Abstract

We report here the complete genome sequence of the Vibrio cholerae O1 bv. El Tor Ogawa strain V060002, isolated in 1997. The data demonstrate that this clinical strain has a single chromosome resulting from recombination of two prototypical chromosomes.

## GENOME ANNOUNCEMENT

Vibrio cholerae is a waterborne pathogen that causes the fatal diarrheal disease cholera. Of the more than 200 serogroups of V. cholerae, O1 and O139 are associated with epidemic and pandemic cholera and with the major virulence determinant cholera toxin (1, 2) produced by the filamentous bacteriophage CTXϕ (3). Serogroup O1 comprises two biotypes, classical and El Tor. The classical biotype caused the sixth and probably earlier cholera pandemics, whereas the El Tor biotype is responsible for the current seventh cholera pandemic (4).

The genome of V. cholerae is split into two circular chromosomes (chr1 and chr2) (5, 6), a feature common in the family Vibrionaceae (7, 8). However, recent genomic studies on V. cholerae isolates have revealed two non-O1/non-O139 strains, each with a single chromosome (9–11). It has also been reported that V. cholerae O1 strains with single chromosomes can be generated by genome engineering (12) or spontaneously isolated as suppressors of lethal mutations that disrupt the replication of chr2 (13, 14).

The sequenced O1 biovar El Tor Ogawa strain V060002 was isolated in 1997 from a patient who traveled to Indonesia, and the strain has been used in our laboratory as a model for studying regulatory mechanisms of chitin-induced natural transformation (15–18). Genomic DNA was extracted with the DNeasy blood and tissue kit (Qiagen) following the manufacturer’s instructions. A 20-kbp library for P6-C4 chemistry was prepared using the RS II SMRTbell template preparation kit version 1.0 (PacBio) and sequenced with the P6 version 2 single-molecule real-time (SMRT) sequencing platform (PacBio). Sequencing reads were assembled *de novo* using the Hierarchical Genome Assembly Process version 3 (HGAP3) (19) with a mean sequence coverage of 196.55-fold. This assembly was corrected with the Quiver consensus algorithm to obtain a high-accuracy genome assembly (19). The contig was further corrected using Pilon version 1.22 (20), and paired-end short reads (300-mer × 2) were obtained from the MiSeq platform (Illumina).

The generated sequence assembly unexpectedly yielded a single circular chromosome with a genome size of 4,057,041 bp and a GC content of 47.5%. The size and number were verified by pulsed-field gel electrophoresis of the intact chromosome of strain V060002 (data not shown). Comparison of the genome sequences of V060002 and the O1 model strain N16961 (5) revealed that a single chromosome of V060002 was generated by recombination of highly homologous insertion sequence elements shared by chr1 and chr2 (99% identity, corresponding to *vc1789* to *vc1790* on chr1 and *vca0791* to *vca0792* on chr2 of N16961). It should be noted that these recombination sites are identical to those of a representative chromosome fusion spontaneously isolated from N16961 with a null mutation of the *dam* gene (14), which is essential for chr2 replication (21).

Annotation of the V060002 genome using the DDBJ Fast Annotation and Submission Tool (DFAST) (22) identified 3,560 coding sequences, 28 rRNA sequences, and 98 tRNA sequences. Strain V060002 also carried well-known gene clusters associated with pathogenesis (23–26), as well as two copies of the CTXφ prophage.

More detailed genomic and phenotypic analyses of this naturally occurring V. cholerae O1 strain with a single chromosome will be presented in future publications.

### Accession number(s).

The annotated chromosome has been deposited in DDBJ/GenBank under the accession number AP018677.

## References

[1] Singh DV, Matte MH, Matte GR, Jiang S, Sabeena F, Shukla BN, Sanyal SC, Huq A, Colwell RR 2001 Molecular analysis of *Vibrio cholerae* O1, O139, non-O1, and non-O139 strains: clonal relationships between clinical and environmental isolates. Appl Environ Microbiol 67:910–921. doi:10.1128/AEM.67.2.910-921.2001. 11157262 PMC92666

[2] Kaper JB, Morris JG, Levine MM 1995 Cholera. Clin Microbiol Rev 8:48–86. 7704895 10.1128/cmr.8.1.48 PMC172849

[3] Waldor MK, Mekalanos JJ 1996 Lysogenic conversion by a filamentous phage encoding cholera toxin. Science 272:1910–1914. doi:10.1126/science.272.5270.1910. 8658163

[4] Harris JB, LaRocque RC, Qadri F, Ryan ET, Calderwood SB 2012 Cholera. Lancet 379:2466–2476. doi:10.1016/S0140-6736(12)60436-X. 22748592 PMC3761070

[5] Heidelberg JF, Eisen JA, Nelson WC, Clayton RA, Gwinn ML, Dodson RJ, Haft DH, Hickey EK, Peterson JD, Umayam L, Gill SR, Nelson KE, Read TD, Tettelin H, Richardson D, Ermolaeva MD, Vamathevan J, Bass S, Qin H, Dragoi I, Sellers P, McDonald L, Utterback T, Fleishmann RD, Nierman WC, White O, Salzberg SL, Smith HO, Colwell RR, Mekalanos JJ, Venter JC, Fraser CM 2000 DNA sequence of both chromosomes of the cholera pathogen *Vibrio cholerae*. Nature 406:477–483. doi:10.1038/35020000. 10952301 PMC8288016

[6] Trucksis M, Michalski J, Deng YK, Kaper JB 1998 The *Vibrio cholerae* genome contains two unique circular chromosomes. Proc Natl Acad Sci U S A 95:14464–14469. doi:10.1073/pnas.95.24.14464. 9826723 PMC24396

[7] Yamaichi Y, Iida T, Park K-S, Yamamoto K, Honda T 1999 Physical and genetic map of the genome of *Vibrio parahaemolyticus*: presence of two chromosomes in *Vibrio* species. Mol Microbiol 31:1513–1521. doi:10.1046/j.1365-2958.1999.01296.x. 10200969

[8] Okada K, Iida T, Kita-Tsukamoto K, Honda T 2005 Vibrios commonly possess two chromosomes. J Bacteriol 187:752–757. doi:10.1128/JB.187.2.752-757.2005. 15629946 PMC543535

[9] Johnson SL, Khiani A, Bishop-Lilly KA, Chapman C, Patel M, Verratti K, Teshima H, Munk AC, Bruce DC, Han CS, Xie G, Davenport KW, Chain P, Sozhamannan S 2015 Complete genome assemblies for two single-chromosome *Vibrio cholerae* isolates, strains 1154-74 (serogroup O49) and 10432-62 (serogroup O27). Genome Announc 3(3):e00462-15. doi:10.1128/genomeA.00462-15. 25977434 PMC4432340

[10] Chapman C, Henry M, Bishop-Lilly KA, Awosika J, Briska A, Ptashkin RN, Wagner T, Rajanna C, Tsang H, Johnson SL, Mokashi VP, Chain PSG, Sozhamannan S 2015 Scanning the landscape of genome architecture of non-O1 and non-O139 *Vibrio cholerae* by whole genome mapping reveals extensive population genetic diversity. PLoS One 10:e0120311. doi:10.1371/journal.pone.0120311. 25794000 PMC4368569

[11] Xie G, Johnson SL, Davenport KW, Rajavel M, Waldminghaus T, Detter JC, Chain PS, Sozhamannan S 2017 Exception to the rule: genomic characterization of naturally occurring unusual *Vibrio cholerae* strains with a single chromosome. Int J Genomics 2017:8724304. doi:10.1155/2017/8724304. 28951866 PMC5603330

[12] Val M-E, Skovgaard O, Ducos-Galand M, Bland MJ, Mazel D 2012 Genome engineering in *Vibrio cholerae*: a feasible approach to address biological issues. PLoS Genet 8:e1002472. doi:10.1371/journal.pgen.1002472. 22253612 PMC3257285

[13] Val M-E, Marbouty M, de Lemos Martins F, Kennedy SP, Kemble H, Bland MJ, Possoz C, Koszul R, Skovgaard O, Mazel D 2016 A checkpoint control orchestrates the replication of the two chromosomes of *Vibrio cholerae*. Sci Adv 2:e1501914. doi:10.1126/sciadv.1501914. 27152358 PMC4846446

[14] Val M-E, Kennedy SP, Soler-Bistué AJ, Barbe V, Bouchier C, Ducos-Galand M, Skovgaard O, Mazel D 2014 Fuse or die: how to survive the loss of Dam in *Vibrio cholerae*. Mol Microbiol 91:665–678. doi:10.1111/mmi.12483. 24308271

[15] Yamamoto S, Morita M, Izumiya H, Watanabe H 2010 Chitin disaccharide (GlcNAc)_2_ induces natural competence in *Vibrio cholerae* through transcriptional and translational activation of a positive regulatory gene *tfoX^VC^*. Gene 457:42–49. doi:10.1016/j.gene.2010.03.003. 20302923

[16] Yamamoto S, Izumiya H, Mitobe J, Morita M, Arakawa E, Ohnishi M, Watanabe H 2011 Identification of a chitin-induced small RNA that regulates translation of the *tfoX* gene, encoding a positive regulator of natural competence in *Vibrio cholerae*. J Bacteriol 193:1953–1965. doi:10.1128/JB.01340-10. 21317321 PMC3133033

[17] Yamamoto S, Mitobe J, Ishikawa T, Wai SN, Ohnishi M, Watanabe H, Izumiya H 2014 Regulation of natural competence by the orphan two-component system sensor kinase ChiS involves a non-canonical transmembrane regulator in *Vibrio cholerae*. Mol Microbiol 91:326–347. doi:10.1111/mmi.12462. 24236404

[18] Yamamoto S, Ohnishi M 2017 Glucose-specific enzyme IIA of the phosphoenolpyruvate: carbohydrate phosphotransferase system modulates chitin signaling pathways in *Vibrio cholerae*. J Bacteriol 199:e00127-17. doi:10.1128/JB.00127-17. 28461445 PMC5573088

[19] Chin C-S, Alexander DH, Marks P, Klammer AA, Drake J, Heiner C, Clum A, Copeland A, Huddleston J, Eichler EE, Turner SW, Korlach J 2013 Nonhybrid, finished microbial genome assemblies from long-read SMRT sequencing data. Nat Methods 10:563–569. doi:10.1038/nmeth.2474. 23644548

[20] Walker BJ, Abeel T, Shea T, Priest M, Abouelliel A, Sakthikumar S, Cuomo CA, Zeng Q, Wortman J, Young SK, Earl AM 2014 Pilon: an integrated tool for comprehensive microbial variant detection and genome assembly improvement. PLoS One 9:e112963. doi:10.1371/journal.pone.0112963. 25409509 PMC4237348

[21] Demarre G, Chattoraj DK 2010 DNA adenine methylation is required to replicate both *Vibrio cholerae* chromosomes once per cell cycle. PLoS Genet 6:e1000939. doi:10.1371/journal.pgen.1000939. 20463886 PMC2865523

[22] Tanizawa Y, Fujisawa T, Nakamura Y 2018 DFAST: a flexible prokaryotic genome annotation pipeline for faster genome publication. Bioinformatics 34:1037–1039. doi:10.1093/bioinformatics/btx713. 29106469 PMC5860143

[23] Karaolis DKR, Johnson JA, Bailey CC, Boedeker EC, Kaper JB, Reeves PR 1998 A *Vibrio cholerae* pathogenicity island associated with epidemic and pandemic strains. Proc Natl Acad Sci U S A 95:3134–3139. doi:10.1073/pnas.95.6.3134. 9501228 PMC19707

[24] Jermyn WS, Boyd EF 2002 Characterization of a novel *Vibrio* pathogenicity island (VPI-2) encoding neuraminidase (*nanH*) among toxigenic *Vibrio cholerae* isolates. Microbiology 148:3681–3693. doi:10.1099/00221287-148-11-3681. 12427958

[25] Dziejman M, Balon E, Boyd D, Fraser CM, Heidelberg JF, Mekalanos JJ 2002 Comparative genomic analysis of *Vibrio cholerae*: genes that correlate with cholera endemic and pandemic disease. Proc Natl Acad Sci U S A 99:1556–1561. doi:10.1073/pnas.042667999. 11818571 PMC122229

[26] O’Shea YA, Finnan S, Reen FJ, Morrissey JP, O’Gara F, Boyd EF 2004 The *Vibrio* seventh pandemic island-II is a 26.9 kb genomic island present in *Vibrio cholerae* El Tor and O139 serogroup isolates that shows homology to a 43.4 kb genomic island in *V. vulnificus*. Microbiology 150:4053–4063. doi:10.1099/mic.0.27172-0. 15583158

